# Decellularized Extracellular Matrix for Tissue Engineering (Review)

**DOI:** 10.17691/stm2022.14.3.07

**Published:** 2022-05-28

**Authors:** E.V. Isaeva, E.E. Beketov, N.V. Arguchinskaya, S.А. Ivanov, P.V. Shegay, А.D. Kaprin

**Affiliations:** Senior Researcher, Laboratory of Biomaterials and Tissue Constructions; A. Tsyb Меdical Radiological Research Centre — Branch of the National Medical Research Radiological Centre of the Ministry of Health of the Russian Federation, 10 Zhukova St., Obninsk, 249036, Russia; Head of the Laboratory of Biomaterials and Tissue Constructions; A. Tsyb Меdical Radiological Research Centre — Branch of the National Medical Research Radiological Centre of the Ministry of Health of the Russian Federation, 10 Zhukova St., Obninsk, 249036, Russia; Junior Researcher, Laboratory of Biomaterials and Tissue Constructions; A. Tsyb Меdical Radiological Research Centre — Branch of the National Medical Research Radiological Centre of the Ministry of Health of the Russian Federation, 10 Zhukova St., Obninsk, 249036, Russia; Director; A. Tsyb Меdical Radiological Research Centre — Branch of the National Medical Research Radiological Centre of the Ministry of Health of the Russian Federation, 10 Zhukova St., Obninsk, 249036, Russia; Professor, Department of Oncology and Radiology and Nuclear Medicine; Peoples’ Friendship University of Russia, 6 Miklukho-Maklaya St., Moscow, 117198, Russia; Head of the Center of Innovative Radiological and Regenerative Technologies; National Medical Research Radiological Center of the Ministry of Health of the Russian Federation, 4 Koroleva St., Obninsk, 249036, Russia; Professor, Academician of the Russian Academy of Sciences, General Director; National Medical Research Radiological Center of the Ministry of Health of the Russian Federation, 4 Koroleva St., Obninsk, 249036, Russia Head of the Department of Urology and Operative Nephrology with the Course of Oncourology of the Medical Institute; Peoples’ Friendship University of Russia, 6 Miklukho-Maklaya St., Moscow, 117198, Russia;

**Keywords:** tissue engineering, decellularized extracellular matrix, dECM, extracellular matrix remodeling, decellularization

## Abstract

In recent years, decellularized tissues have evolved into a new, full-fledged platform for the creation of tissue-engineered constructions. Extracellular matrix (ECM) of each tissue provides a unique tissue-specific microenvironment for resident cells with the structure and biochemical signaling required for their functioning. The decellularized ECM (dECM) has been established to influence cell differentiation.

The review provides recent data on the composition and functions of the ECM, methods for obtaining decellularized tissues, and their application in tissue engineering depending on their physical form (scaffold, powder, or hydrogel). The effect of the matrix source, decellularization and sterilization techniques on dECM composition has been considered. Regulatory mechanisms of cell differentiation by the extracellular matrix are discussed. Differences in the protein composition of the native and decellularized materials are presented. Application of dECM in the bioink composition for regeneration of various tissues using bioprinting technologies is also considered.

It has been concluded that successful application of dECM in tissue engineering and regenerative medicine requires a permanent and biologically suitable dECM source, optimized tissue decellularization protocols, improved mechanical properties of dECM-derived bioinks, and prevention of immunological reaction of the organism.

## Introduction

In recent years, decellularized tissues and organs have turned into a new full-fledged platform for creation of tissue-engineered constructs (scaffolds) along with naturally derived and synthetic hydrogels and bioinert polymers [1, 2]. An extracellular matrix (ECM) is the main product of decellularization. It may not only serve as a physical scaffold into which the cells are built-in, but is also capable of regulating many cellular processes including growth, migration, differentiation, homeostasis, and morphogenesis [3-6]. ECM of each tissue creates a unique tissue-specific microenvironment for the resident cells providing them with the structure and biochemical signals necessary for their functioning within the concrete tissue [7]. Thus, we may expect that decellularized tissues must exert some influence on cell differentiation depending on the tissue from which the material was fabricated. Actually, the application of decellularized tissues (dECM, to be more exact) may not so much be a highly specialized technique of creating tissue scaffolds but a meaningful direction of medical and biological investigations in the field of regenerative medicine and tissue engineering to control cell behavior and scaffold development into a fully functional tissue [2].

Despite the evident advantages of using dECM as the main material or a supplement to scaffolds, there is a series of obstacles restricting its applications: e.g., the extent of active ingredients remained in the decellularized tissue may be different, it may also vary in the course of subsequent treatment, sterilization, conservation, and other processes. Presently, the detailed mechanism of interaction of ECM and cells *in vivo* is not clear. The exact dECM composition which promotes a definite cell behavior, tissue regeneration, and angiogenesis has not as yet been known, the appropriate cellular and molecular mechanisms are also worth exploring along with immune rejection *in vivo* [8].

**The aim of this review** is to systematize the information on the dECM composition and functions, its effect on cell differentiation, methods of fabrication, and application in tissue engineering.

## Extracellular matrix properties

### Constituents and functions

All tissues and organs contain cells and non-cellular components, which form well-organized networks, i.e. ECM. ECM consists of a great number of matrix macromolecules, whose exact composition and certain structure vary from tissue to tissue. In mammals, ECM consists of about 300 proteins known as the core matrisome [9]. The base constituents of ECM are proteins with a fibrous structure such as collagens, elastin, fibronectin, laminins, glycoproteins, proteoglycans, and glycosaminoglycans (GAG) which represent acidic hydrated molecules.

Depending on the tissue (organ), different types of collagen are present in various proportions [10]. In the majority of tissues, type I and II collagens are the main constituents of ECM. They are connected with other collagens, as well as with ECM proteins and proteoglycans forming large fibrillar structures. These multimolecular structures, filling the space between the cells, create a complicated three-dimensional matrix network [6]. Tissue-specific networks modulate migration of the cells and transmission of biomechanical forces [11]. Collagen fibrils impart tensile strength and limit tissue stretchability. Aiyelabegan and Sadroddiny [12] have reported that other physical characteristics of ECM such as location, insolubility, stiffness, and porosity, which determine the mechanical behavior of each tissue and the cells composing it, result from the ECM composition.

Proteoglycans (glycoprotein subclass) such as aggrecan, versican, perlecan, and decorin represent heart-shaped proteins with the attached side GAG chains, which are spread out among collagen fibrils. Proteoglycans fill the intercellular interstitial space and perform hydration functions binding water within the tissue. GAG, especially heparin sulphates, also bind many growth factors isolating them in ECM. Other glycoproteins such as laminins, elastin, fibronectins, thrombospondins, tenascins, and nidogen fulfill diverse functions. Elastin is a key molecule of tissue elasticity; laminins are a basic component of the basement membrane; fibronectin is a glycoprotein performing simultaneously several functions: cellular adhesion, migration, growth, and differentiation [10]. Apart from their role in the ECM assembly, glycoproteins are also involved in the ECM — cell interaction acting as ligands for the receptors of the cellular surface such as integrins. Upon the whole, glycoproteins act as a reservoir of growth factors, which are linked to ECM and are released after proteolysis. At the same time, in the process of glycoprotein cleavage, fragments with the functions different from those of the initial full-sized protein may be formed [13].

In some works [14, 15], ECM has been shown to play an important role in the fetal development and further fate of the stem precursor cells, it also impacts the shape, survival, and proliferation of the cells. The functional value of ECM is illustrated by a wide spectrum of tissue defects or, in severe cases, of embryonic lethality, caused by mutation in the genes, which encode the ECM components. The studies of the function loss have also shown the importance of ECM proteins in the development processes since the deletion in the genes of some ECM, such as fibronectin and collagens, often leads to the lethal outcomes for embryos [16].

Theocharis et al. [17] divide ECM into two main types, which differ in the composition and structure: interstitial and pericellular. The interstitial, or intercellular matrix, surrounds the cells while the pericellular one is in close contact with it. Basement membrane, for example, is referred to the latter type of the matrix. The membrane is located on the interface between the parenchyma and connective tissue, which provides a laminated anchor layer for parenchymal cells to hold them together preventing their rupture. The basement membranes consist of four main components: type IV collagen, laminin, nidogen/entactin, and perlican [18]. Cells, built in ECM, interact with this macromolecular network via their superficial receptors such as integrins, discoid domain receptors, proteoglycans on the cell surface, and receptors of hyaluronic acid CD44. All types of the cells (fibroblasts, epithelial, immune, and endothelial) synthetize and secrete matrix macromolecules under the control of multiple signals participating thereby in ECM formation. Various growth factors, cytokines and chemokines, secreted C-type lektins, galectins, semaphorins, plexins, and enzymes modifying ECM which are involved in crosslinking (for example, transglutaminase, lysiloxidase, and hydroxylase), not being part of the matrisome, are important in ECM remodeling [13]. They are accumulated in the matrix by binding to the concrete molecules and can be released and act at the definite stages of the physiological development [16, 19].

ECM has been established to be of crucial importance for normal homeostasis [14] and its remodeling determines regulation of morphogenesis of the intestine and lungs, mammary and salivary glands [13, 20]. Disorders in the regulation of the composition, structure, stiffness, and amount of ECM, as well as the processes of remodeling are associated with pathological conditions and may aggravate the disease progression. For example, abnormal ECM deposition and stiffness are observed in fibrosis and cancer [4], while excessive degradation is associated with osteoarthritis [21]. Considerable ECM alterations occur in the following pathologies: arteriosclerosis, autoimmune and inflammatory diseases. Dystrophic epidermolysis bullosa, a genetically determined skin disease, caused by abnormal functioning of type VII collagen, is referred to the diseases of this type [22]. Presently, the microenvironment, in which diseases are progressing, is believed to be not less important than the cell populations engaged in the development of pathological conditions [17]. The structure, interaction, and functions of ECM components are presented in detail in the works [14, 17, 20].

### Effect of extracellular matrix on cell differentiation

Decellularized materials have been proved to influence cell differentiation. Today, lungs, liver, muscles, kidneys, heart, central nervous system, adipose tissue, cartilages, and other decellularized tissues have shown the signs of positive impact on cell differentiation under the laboratory conditions [2]. Possible mechanisms of this process were investigated [2, 23–26]. Thus, Ragelle et al. [27] have shown that each tissue has a unique composition of ECM, which determines the way of cell differentiation. Tissue specificity of ECM is formed in the process of histo- and organogenesis and maintained afterwards. Naba et al. [28] have revealed that apart from structural proteins, enzymes take an active part in matrix building and renewal, primarily, matrix metalloproteinases which cause polymerization and maturation of protein fibrils, and also proteolysis of ECM proteins. Thus, ECM represents tissue-specific network of protein polymers, the composition of which is determined by its stiffness, elasticity, porosity, extent of hydration, and the ability to interact with the cells, extracellular vesicles, and biologically active molecules such as growth factors. All these ECM properties determine its ability to regulate cell activity and to play an important role in their fate, in particular in the stem cells of the postnatal organism [2, 13, 24].

ECM remodeling effect has been found by Lu et al. [20] to be an important mechanism by which it is possible to regulate cell differentiation including the processes such as creation and maintenance of stem cell niches, branching morphogenesis, angiogenesis, bone formation, and wound healing. Renewal of many human organ tissues takes place owing to proliferation and differentiation of tissue-specific stem cells, which are located in specialized “niches” in the tissues. Potentially, ECM must promote differentiation of the stem cells into the cells of the tissue from which it was isolated, so it possesses tissue specificity in maintaining a definite niche for the cells [2]. ECM can provide an adequate cellular microenvironment, proliferation, polarization, and migration of the cells. Yamashita et al. [29] have demonstrated that the potential mechanism by which ECM regulates the biology of the stem cells lies in supporting cell polarity, orientation of the mitotic spindle, and asymmetric cell division. ECM binds growth factors and interacts with cellular surface receptors to direct signal transmission and regulate gene transcription controlling important morphological and physiological functions [30]. Thus, both the composition and physical properties of ECM influence self-renewal, proliferation, and differentiation of the stem cells [24].

## Fabrication of decellularized extracellular matrix

Decellularization is a process of removing the cells and genetic material from ECM preserving its structural, biochemical, and biophysical properties as much as possible [10]. Apart from removing the cellular content [31] and nuclear material [32, 33] retaining intact the native ECM structure [34], the decellularization procedure also requires the removal of potential impurities and detergents [34]. Successful fabrication of dECM depends on the type of the initial tissue, methods of decellularization, and subsequent final processes, for example, final sterilization or chemical modification. Manipulations and concrete reagents of each manufacturing stage have a significant effect on physical and biochemical properties of the matrix, as well as on the subsequent cellular response and, as a consequence, on the result of tissue remodeling [7].

### The role of the decellularized extracellular matrix source

As is well known, dECM is obtained by decellularization and processing of the initial tissues harvested from people (allogenic ECM) or animals (xenogenic ECM) [35]. Porcine organs are used most frequently for dECM fabrication [36, 37]. Cattle, goats, and rats may also serve as a tissue source [38]. Rat and cattle dECM is widely used for investigations but not in clinical practice [36, 37]. Human dECM may be obtained, for example, from the adipose tissue collected during liposuction, or donated organs including cadaveric material [39]. Cramer and Badylak [7] have found that molecules composing ECM are highly conservative in mammals. This is one of the reasons why xenogenic dECM does not cause any adverse inflammatory reaction when implanted into a human [7]. Among the proteins, the most evolutionarily conservative are the proteins of the basement membrane such as laminin and type IV collagen are [40]. High interspecific homology is also observed for other ECM components including collagens, fibronectin, GAG, and growth factors [7]. At the same time, Dzobo et al. [37] believe that tissue ECM of the same animal species may have differences depending on the age and gender. Donor’s age of the initial tissue influences not only ECM mechanical properties but, what is more important, the composition of the dECM obtained [41-44]. Wang et al. [45] have found that fetal and newborn tissue ECM is especially rich with GAG such as hyaluronic acid and fibronectin. The content of laminin [46], elastin [45, 46], and growth factors [43] decreases with age. The collagen of young animals contains less lateral cross-links than that of an adult organism, which is the factor contributing to faster degradation of the young-animal ECM than of the grown-up animals [43]. The authors [47, 48] paid attention to the fact that xenogenic sources of dECM may be potentially dangerous as they may carry the diseases common for people and animals.

### Decellularization agents and their effect on the decellularized extracellular matrix composition

The existing methods of decellularization are presented in many studies [10, 35, 36, 49–52]. Different decellularization agents are used depending on the number and type of cells, tissue thickness, lipid content, etc. [32, 36]. Broadly, they can be divided into four groups: 1) chemical including acids and bases, detergents, hypo- and hypertonic solutions, spirits, and solvents; 2) biological including enzymes, nucleases and chelating agents, in particular; 3) physical including temperature, osmotic pressure, electroporation, and direct force impact; 4) combination of the two or all mentioned methods [10, 32, 34–36, 49–52].

Treatment may last from one day to a week or longer. Some agents are able to influence the dECM composition. For example, ammonium hydroxide, sodium dodecyl sulfate, and triton X-100 (standard chemical reagents for decellularization) remove or damage ECM components [32]. Processing with acid or alkali including ammonium hydroxide and sodium hydroxide may result in the elimination of growth factors and affect the dECM strength (organ scaffold). At the same time, one of the advantages of using acidic and alkaline treatment is sterilization of the final product. Triton X-100 was shown [35, 53] to reduce the amount of fibronectin and laminin in dECM and to damage the matrix ultrastructure in addition to GAG elimination. Ammonium hydroxide and sodium dodecyl sulfate cause collagen damage [53-57]. Sodium dodecyl sulfate has been found to affect negatively the growth factors [32]. The possibility to use the latter reagent for dense tissues and organs may be considered to be its merit.

Enzymatic decellularization is implemented with nucleases (DNase and RNase) and proteases (trypsin, dispase). However, an excessively long exposure of the tissue and organ to trypsin can result in the damage to the matrix structure and removal of proteins such as collagen, laminin, fibronectin, and elastin as is reported in the literature [32, 49, 53]. The same negative effect on the collagen is produced by nucleases; besides, there are difficulties in their elimination from the tissue and organ.

Physical decellularization, despite the less damage to the ECM structure, may lead to incomplete removal of the cellular debris, which in turn may cause immune reactions, especially in case of using dECM for transplantation.

### Decellularization procedure

There are four methods of treating organs and tissues with the agents: application of perfusion, pressure gradient, supercritical fluid, and also immersion and agitation [36]. Large organs are usually decellularized by way of perfusion [58]. If it is not necessary to retain organ scaffold, as, for example, in bioink fabrication, tissues may be cut or crushed into fine pieces [59, 60] which are subsequently processed with a decellularization agent by shaking during different periods of time (from hours to days, and sometimes for weeks) [32, 36, 53, 61, 62]. However, full dissolution is one of the main drawbacks of using small pieces of the tissue or organ. In general, differences in the ECM composition require various protocols of decellularization and treatment depending on the type of organism, tissue, donor’s age, and the anatomical area.

Decellularization is a long-term process which requires close control by the researcher and a large percentage of operations performed manually. Therefore, fabrication of a large amount of functional dECM is difficult. Choudhury et al. [34] believe that automation of the process and integration of various protocols will make it possible to reduce considerably the time spent for decellularization and increase the quantity of dECM. This will promote a wider use of the matrix in regenerative medicine. The authors describe modern systems employed for decellularization. Unfortunately, presently, the majority of the described automatic systems designed to optimize the process of decellularization are used by a limited number of researchers in the companies where these methods have been developed.

### Sterilization of the decellularized extracellular matrix

The final stage of treatment is sterilization of dECM. Ethanol and peracetic acid are used most frequently for these purposes [37]. Gamma radiation at the doses ranging from 1000 to 10,000 Gy (3000 Gy on average) is also used for dECM sterilization [63, 64]. Gaseous ethylene oxide and carbon dioxide are also applied as an alternative technique [63, 65, 66]. One of the main problems of the sterilization stage is connected with the possible changes in the composition of the fabricated dECM [37]. This applies especially to the effect of gamma radiation [64].

### Criteria of decellularizarion completeness

Immunogenicity, thrombogenicity, and ECM alteration remain the main drawbacks of all protocols. Successful decellularization implies achievement of two conditions: removal of the cellular material and retention of matrix functionality [10]. There are no universal and commonly accepted standards to assess decellularization completeness adequately. At present, different research groups are using various evaluation methods [34]. Crapo et al. [32] have proposed minimal criteria to assess the removal of the residual DNA and genetic material which, in their opinion, would be sufficient to verify decellularization. These criteria imply the following: 1) dECM must contain less than 50 ng of DNA per 1 mg of dry ECM mass; 2) the length of DNA fragments should be less than 200 pairs of nucleotides; 3) tissue sections stained with DAPI or hematoxylin and eosin must not contain traces of genetic material. Gilpin and Yang [67] believe that the process of decellularization may be considered successful if at least 90% of the host’s DNA is removed.

There are some other methods of identifying the completeness of decellularization: a qualitative assessment of residual detergents, histological and biochemical investigations for the presence of ECM components such as collagen and GAG [34, 52]. Although these criteria may be useful for evaluation of the extent of cell removal, further explorations are required to define a threshold for the induction of the immune response after the implantation into the host’s organism [10].

Evaluation of the dECM functionality must include compositional, structural, and mechanical analysis in order to assess the changes occurred in ECM [10]. Presence of collagen, elastin, laminins, fibronectin, and GAG after the decellularization process is of key importance for maintaining the adequate dECM functionality. Mechanical properties, such as tensile strength, modulus of elasticity, modulus of viscosity, stiffness, or yield strength are also of great interest. Another parameter, which should be taken into consideration, is anisotropic or isotropic tissue characteristics since they may to some extent control the orientation of the reseeded cells [10].

## Treatment of the decellularized extracellular matrix

Ultimately, decellularization is aimed at obtaining two different products: a full organ scaffold or a loose dECM tissue. When the whole organ is cellularized, its three-dimensional structure is retained including the vascular network. Its capabilities may be potentially realized during subsequent recellularization [68-70]. The other product is a loose dECM tissue, which represent purified and sterilized ECM isolated from the organ [34]. It may be used for obtaining matrix powder and/or bioinks.

Decellularized matrix itself is the basis for recellularization which may be performed before and after implantation [71]. Implanted naked scaffolds from dECM will be infiltrated by the recipient’s native cells, which with time will replace the decellularized matrix with a newly-formed ECM [72-74]. Moreover, Etnel et al. [75] have shown that the decellularized transplants themselves may give satisfying clinical results and, consequently, the question about repopulation of the decellularized matrix with the appropriate cells remains open [10]. The dECM material, which retains its initial structure, possesses some advantages: intact vascular network, exact shape of the derived tissue, and retained mechanical strength. Although such dECM forms undergo a less number of treatment stages, their clinical application is somewhat limited since the structure hinders conformational adaptation [76].

### Powder fabrication

Decellularized ECM can be crushed into powder, which is subsequently used in the form of particles, or is dissolved enzymatically and used as a fluid or gel. Enzymatic digestion of such powder requires an acidic medium, therefore, hydrochloric acid or peracetic acid and pepsin are usually employed for dissolving [76]. For subsequent usage, the acidic solution is neutralized up to physiological pH [76, 77]. The powder and constructions based on the dECM liquid suspensions and gels may fill the areas of space occupying injuries and conform to the contours of the native tissues. The adaptive powder form and its soluble form make it possible to perform minimally invasive implantation, e.g. injection. In contrast to constructions with a fixed structure, which have a tendency to shrinking, dECM in the form of a powder and solutions retains the shape it has taken [78].

Preparation of the dECM powder includes an extensive treatment consisting of freezing, lyophilization, and milling. Potentially, it alters the biological integrity of dECM. The treatment stages may influence the composition, mechanical strength, and dECM ultrastructure [79]. Conceivably, dECM components are destroyed at each manufacturing stage and the process may in its turn change the host’s reaction to implantation *in vivo* [80]. Despite the reports about the constructions demonstrating desired bioactivity, tissue-specified parameters for the optimal fabrication of the powder are not defined [76]. To optimize the application of the dECM powder, the following factors should be taken into consideration but not limited by them: the amount and concentration of the powder used in the constructions; the size and morphology of particles; powder dissolution and dECM cross-linking. All methods and protocols should be reproducible if constructions are used under clinical conditions [76].

### Fabrication of bioinks

With the development of bioprinting technologies, dECM-based hydrogels received a new application in the form of bioinks. Kabirian and Mozafari [35] define bioinks as the main building blocks of the printed constructions playing a decisive role in supporting and providing appropriate medium for the incorporated cells. The requirements, which bioinks should meet, are presented in many works [57, 81–85]. A combination of bioprinting technology and bioinks with dECM is a promising and logic approach to scaffold creation [51].

Decellularized ECM hydrogels consist of functional, structural, and signaling molecules such as collagen, laminin, fibronectin, GAG, and growth factors, which may retain in dECM. Therefore, dECM-derived bioinks represent a biomaterial demonstrating the highest degree of similarity with the native tissue [51]. dECM may be used as a single hydrogel component or with the application of cross-linking agents and creation of composites with supplemental materials [59, 62, 78, 86–94]. A common method of fabricating hydrogels with dECM as a single element in the material includes lyophilization, milling, and digestion of dECM with pepsin. Prior to cell addition, pH is brought to neutral values [91, 92]. Hydrogels are formed at the temperature of +37°С [77]. The disadvantage of such gels is their low viscosity, which makes them poorly suited for printing [36]. Composite hydrogels may be fabricated by photo cross-linking [84, 92, 95], chemical cross-linking [96, 97], or by the addition of other materials to improve mechanical and rheological characteristics [89, 93].

## Differences in the protein composition between the native and decellularized extracellular matrix

Proteins are the most important ECM components responsible for its biomimetic properties. However, the protein composition will be different before and after decellularization. The protein profile of some porcine tissues is presented by Choudhury et al. [36]. Visscher et al. [95] have noted a significant alteration of the proteome of the auricle-derived decellularized elastic porcine cartilage in comparison with the native tissue. The collagen and GAG content in the decellularized cartilage tissue was considerably low, elastin, the main component of the elastic cartilage, was absent. The proteomic analysis has found 683 unique proteins contained only in the native cartilage, 21 proteins only in the decelularized cartilage, and 412 proteins in both the native and decellularized cartilages. The total amount of proteins identified in both tissues was equal to 1063±54 for the native and 427±129 for the cellularized cartilages.

At the same time, Nagao et al. [98] have reported that during decellularization of the human renal cortical layer, the majority of the native matrix proteins such as type IV collagen, laminin, and heparin sulfate proteoglycan (HSPG), as well as their isoforms retained in the same proportions as in the normal kidneys. All six α-chains of type IV collagen have been detected. The decellularization process preserved not only the COL4A1 and COL4A2 chains, which are present everywhere in all basement membranes, but also the COL4A3 and COL4A5 chains, which are located only in specialized basement membranes within the kidney glomeruli. The amount of type I collagen was 20.1±2.3% of all proteins. The HSPG protein was identified despite the use of a strong anion detergent; type XVIII collagen, the HSPG component of the renal cortex basement membranes, was also available in the amount of about 1%. Other matrix components such as vitronectin, fibrinogen, and elastin were detected in 2.4, 0.4, and 0.3%, respectively [98].

## Application of decellularized extracellular matrix in tissue engineering

At present, numerous tissues and organs are decellularized for practical application of ECM, and dECM has already been successfully employed in clinical practice for regeneration of various tissues. The biological materials including mammal dECM are used in the tissue engineering of the cardiac muscle [86, 87, 90, 99–101], cartilage [92, 93, 95, 100–105], tendons [106, 107], liver [59, 87, 91, 94], skin [62, 88, 108, 109], cornea [110], respiratory tracts [70], adipose tissue [100, 101, 111], bones [112, 113], brain [96], kidneys [98]. In the majority of these works, dECM hydrogel is a constituent part of bioinks, while scaffold were obtained by 3D bioprinting.

Some interesting works on this topic should be noted. Pati et al. [100] described the method of bioprinting cell-loaded constructions with bioinks based on dECM derived from the human adipose tissue, porcine cartilaginous and cardiac tissue. The authors observed differentiation of the human stem cells and rat myoblasts in the necessary direction and formation of the appropriate tissue without any supplemental exogenous growth factors. Although it has been reported that 3% dECM-based gel retains the shape after printing, the authors nonetheless used an additional supporting scaffold from polycaprolactone when constructions with the matrix from cartilaginous and adipose tissue were fabricated.

Fabrication of bioinks from dECM has been described by Kim et al. [110], who used cornea as a source of ECM. The developed bioinks had similar amounts of collagen and GAG in comparison with the native cornea and possessed the desired transparency to provide vision.

The efficacy of the soluble dECM fraction has been shown by Rothrauff et al. [105]. Gelatin metacryloyl-based bioinks supplemented with a soluble dECM fraction from the calf meniscus accelerated chondrogenic differentiation of human mesenchymal stem cells when cultivated in the chondrogenic medium, which was confirmed by the gene expression analysis and increased formation of sulphated GAG. However, the scaffold was fabricated here using molding technique, i.e. without 3D printing.

The efficacy of the approach has been demonstrated in the study of Zhang and Dong [114] where they assessed the properties of bioinks based on the silk fibroin and dECM derived from the goat cartilaginous tissue. The bioinks containing bone marrow stem cells and supplemented with the transforming growth factor β3 (TGF-β3) promoted chondrogenic differentiation of the stem cells, while the scaffold itself had a good strength and high biodegradation rate.

Some works describe application of dECM powder mainly for regeneration of the cartilaginous tissue. Thus, Yin et al. [104] have found that bone marrow stem cells proliferated actively on the surface of dECM particles and differentiated into mature chondrocytes 21 days later without any exogenous growth factors. With the increase of cultivation time, functional aggregates of the micro-organic cartilaginous structures were being formed which, after being implanted into the defects of the rats’ knee cartilaginous tissue, contributed to faster and better healing relative to the control groups where the defects were treated only with dECM particles or only with fibrin glue [104].

Barthold et al. [115] added dECM powder to the hyaluronic acid hydrogel and observed migration of chondrocytes to the granules. Thitiset et al. [112] have shown that demineralized bone powder (250–500-μ particle size) gives the necessary osteoinductive stimulus to human periosteal cells without supplementary exogenous growth factors. Almeida et al. [102] have developed injectable fibrin hydrogel functionalized with cartilage-derived dECM microparticles and TGF-β3 as a potential therapeutic means for regeneration of the joint cartilage. After 28 days of cultivation *in vitro*, composites fibrin−dECM supplemented with TGF-β3 microscopically resembled a cartilage.

Single works report about the inclusion of dECM powder into the bioink composition for scaffold fabrication. To replace a joint cartilage, the authors used bioinks prepared from the porcine cartilage-derived dECM powder (18% formulation) mixed with the solution of silk fibrin (7%) [30]. Mesenchymal stem cells from the bone marrow were seeded from above after 3D printing procedure, which does not allow these bioinks to be considered true since the cells should be incorporated into them before printing. In another study, the composition based on the hyaluronic acid (3 mg/ml), gelatin (37.5 mg/ml), and fibrinogen (3 mg/ml) supplemented with porcine liver-derived dECM powder with a particle size of ~13.4 μm played the role of bioinks [94]. The results have shown good mechanical properties and, consequently, suitability for 3D printing relative to the bioinks from dECM hydrogel, and also high biocompatibility.

We listed only part of the published investigations characterizing the main trends in the application of dECM in tissue engineering. The published data allows us to conclude that the effect of dECM on cell differentiation is beyond doubt. However, the extent to which each tissue can control the cell lines is different. The heart, liver, and adipose tissue demonstrate the ability to differentiation in the presence of only dECM without any supplementary factors [59, 87, 89, 91, 100, 101, 114, 116, 117]. In case of other tissues, for example, lung and kidney, dECM only is not enough for differentiation of the stem cells in the desired direction and supplementary stimuli are required to form a new tissue [118, 119].

## Conclusion

Tissue engineered constructions including mammal tissue-derived dECM, may promote favorable processes of tissue regeneration in a wide range of clinical applications. The mechanisms of tissue remodeling by dECM include, among others, degradation and generation of bioactive molecules, recruiting and differentiation of the stem cells and precursor cells as well as modulation of the immune response. These positive results depend crucially on the methods used for dECM fabrication. The issue source, decellularization protocol, and additional treatment stages influence the cellular response and the result of remodeling the scaffold with dECM. The dECM-based bioinks provide a new approach to the creation of biomimetic tissue-engineered constructions.

A constant and biologically suitable source of ECM, optimized protocols for tissue decellularization preventing alterations in matrix composition, improvement of mechanical properties of dECM-based bioinks, and averting immunological reaction of the organism are necessary for successful application of dECM in tissue engineering.
